# Association between Self-Classification of COVID-19 Risk Levels and Adverse Lifestyle Changes among Physically Active Older Adults Following the Coronavirus Outbreak

**DOI:** 10.3390/ijerph19127039

**Published:** 2022-06-08

**Authors:** Pnina Marom, Beth G. Zalcman, Rachel Dankner

**Affiliations:** 1 School of Public Health, Sackler Faculty of Medicine, Tel-Aviv University, Tel-Aviv 6997801, Israel; pninamarom87@gmail.com; 2 Reuth Research and Development Institute, Reuth Rehabilitation Hospital, Tel-Aviv 6772829, Israel; beth.zalcman@reuth.org.il; 3 Unit for Cardiovascular Epidemiology, The Gertner Institute for Epidemiology and Health Policy Research, Sheba Medical Center, Tel Hashomer, Ramat Gan 5262100, Israel

**Keywords:** risk perception, pandemic, health behavior change, physical activity, body weight, smoking

## Abstract

The COVID-19 pandemic has imposed barriers to a healthy lifestyle, especially for older adults who are considered to be at a high-risk of infection. This study examined the associations between negative changes and the self-classification to COVID-19 risk level among physically active older adults who are members of a nationwide health club chain. A cross-sectional digital survey was sent to 19,160 older adults (age ≥ 65). The data collected included information on the subjects’ self-classification to the COVID-19 high-risk group (HRG) and changes in physical activity (PA), body weight, and smoking habits since the outbreak. Logistic regression models were used to investigate the associations between the dependent variables of ‘experienced a negative change’ and the independent variables. Of the 1670 survey respondents, 78.3% classified themselves as COVID-19 HRG. Over half of the respondents reported a reduction in PA hours, 26.6% reported weight gain, and 17.7% of smokers increased their amount of smoking. A self-classification to the HRG was associated with 1.46 (95%CI 1.10–1.93, *p* < 0.009) and 1.67 (95%CI 1.21–2.31, *p* < 0.002) greater odds for reduced hours of exercise and weight gain compared to the not high-risk group, respectively. Decision makers should consider how policies may cause barriers to a healthy lifestyle and develop risk communication strategies to encourage positive health-related behaviors, even during a pandemic.

## 1. Introduction

The 2019 coronavirus disease (COVID-19) has affected people’s lives across the globe. The World Health Organization (WHO) first classified the COVID-19 outbreak as a pandemic in March 2020 [1], and most countries mobilized their resources and took significant steps to slow the ongoing spread of the disease. Many of these steps included emergency regulations that restricted individual mobility and social gatherings [2]. These included restrictions that closed international borders, schools, restaurants, sports clubs, and indoor sports facilities, such as gyms, studios, and swimming pools. Although restrictions such as lockdowns and social distancing might have saved lives, they majorly impacted individuals’ lives over the past year due to both the direct effects on physical and mental health as well as the indirect socio-economic effects [3].

Data gathered since the beginning of the outbreak have shown that COVID-19 constitutes a significant risk for serious illness to older people. Risk perception refers to the individual’s perceived susceptibility to a threat [4]. It has been widely explored in different contexts and has been found to play an important role in affecting both health behaviors [5] and psychological well-being, especially among people who are in quarantine or in high-risk areas [6,7]. Perceived risk was also found to affect the ways people react to the emergency government restrictions [5].

### A Brief Review of COVID-19 Impacts on Health Behaviors among the General Population and Older Adults

During these unforeseen circumstances and modifications in daily life activities, many people experienced changes in their health behaviors due to restricting activities to home spaces, reducing physical activity (PA), and increasing sedentary behavior [8]. Empirical evidence of the impact of the COVID-19 pandemic on health-related behaviors of different groups is still emerging, and findings so far have been mixed [9,10,11,12,13,14,15,16,17,18,19,20,21,22,23]. Some studies have demonstrated that as a result of COVID-19, specifically due to lockdowns and the disruption in everyday life, there has been a dramatic negative shift in lifestyle parameters such as sleep patterns, substance use, exercise, and diet [8]. For instance, Catucci et al. [24] reported a negative effect of the first COVID-19 lockdown in Italy and some European countries on eating habits and PA. Their findings were based on the observation that there has been an increase in food consumption and a reduction in PA with a consequent increase in weight. These negative lifestyle changes may be associated with negative health consequences [8]. Other studies, however, have shown positive trends. One study, which included over 30,000 participants across 31 European countries, found lower alcohol consumption among almost all countries, as people were not able to attend heavy episodic drinking events [25]. Other studies and surveys have found lower rates of smoking, cannabis use, and binge drinking [26,27].

As older adults are disproportionately affected by this disease, many countries implemented strict social isolation to control and prevent deaths [28]. Regarding the impact of the pandemic on older adults’ lifestyle, it could be assumed that older populations would be more susceptible to a negative impact on health behavior due to a reduction in physical movement, poor mental state, changes in appetite, and loneliness [28,29,30,31]. However, reported findings on the COVID-19 pandemic’s impact on older adults are mixed. While some studies showed older cohorts were less likely to report changes in health behaviors compared to the younger cohorts [10,13,15], Visser et al. [23] found a negative impact on the nutrition and PA of Dutch older adults, which may increase the risk of malnutrition, frailty, sarcopenia, and disability. Furthermore, a meta-analysis examining whether PA patterns changed as a result of COVID-19 and to what extent found that of the seven studies related to older adults only three found a significant decrease. Three other studies found mixed results, and one found no change [32]. Other studies showed a decline in physical functioning and well-being, including PA, among older adults during the pandemic [33,34].

A clear need for studies investigating the impact of COVID-19 on lifestyle, especially among older adults has been expressed [8,35]. In Israel, health clubs were opened and closed intermittently, which made it difficult for members to maintain an active lifestyle. Elderly individuals who are also health club members comprise a unique group with special characteristics and needs that have not yet been studied in the context of the pandemic’s impact on health behaviors nor in relation to COVID-19 risk perception. Exploring risk perception, specifically in the form of self-classification to a risk level, could contribute to identifying vulnerable populations among this unique group of older adults and adjusting policies adopted by the governments.

This study aimed to evaluate changes in health behaviors, including exercise habits, body weight, and smoking, that occurred since the COVID-19 outbreak was announced in Israel among older adult (age ≥ 65 years) health club chain members and to examine the associations between negative changes in health behaviors and the self-classification of the COVID-19 risk level.

## 2. Materials and Methods

### 2.1. Study Design and Participants

The study is based on a cross-sectional survey, and participants were physically active adults, 65 years and older, who were members of “Holmes Place”, a health club chain. “Holmes Place” is one of the largest health and fitness club chains in Israel, with 49 nationwide branches, more than 123,000 members of all ages, and 19,160 members ages 65 and older. The clubs offer a wide range of sports facilities and physical activities, including aerobic studios, gyms, swimming pools, and tennis courts.

### 2.2. Procedures

The survey was distributed between 24 July and 2 August 2020 (data collection lasted ten days) during the second wave of the COVID-19 pandemic. During that period, health clubs, fitness centers, gyms, studios, and swimming pools all over the country were shut down for almost a month for the second time since the beginning of the COVID-19 outbreak. At the time of the survey, the only mode of exercise allowed was individual sports in open public spaces, with uncertainty regarding when the clubs would be allowed to reopen.

A text message was sent to the cell phones of all members of the health club chain with a proper phone number containing a brief explanation about the survey and a hyperlink to the questionnaire web page. Before filling out the survey, each participant was required to mark “√” to confirm that s/he was aware that by submitting the survey form they were providing consent to anonymously participate in the study. Respondents could stop answering the survey and leave the questionnaire at any stage. Their answers were saved and reported by clicking on the “send” button provided.

### 2.3. The Survey and Study Variables

For the purpose of this study, a short digitally self-administered questionnaire was designed that aimed to maximize the response rate with the use of simple, easy-to-understand, and focused questions. It consisted of 13 questions relating to socio-demographic data (sex, age, and place of residence), self-classification of COVID-19 risk level, and changes in selected health behaviors since the beginning of the outbreak. Three key lifestyle habits of physically active older adults were surveyed: exercise, body weight, and smoking.

Respondents were asked if they were at a higher risk of developing serious illness from COVID-19 based on the WHO criteria [36]. Respondents were given the information that individuals who are considered to be part of a COVID-19 high-risk group have at least one of the following criteria: age 65 years and older, cardiovascular disease, diabetes, respiratory diseases, diseases affecting the immune system, cancer, smoking, or obesity. The answers given to this question were “yes” or “no”. This question enabled us to generate the main independent variable and to divide the sample into two categories by the self-classification to a COVID-19 risk level: the high-risk group (HRG) and the not high-risk group. Those who answered “no” were classified as “other” since either they do not know they are in a risk group, or they do not perceive themselves as high-risk. For each of the survey’s domains, exercise, body weight, and smoking, respondents were asked to report any positive or negative change in that specific health behavior since the beginning of the pandemic. If a respondent answered “yes” on the first question s/he was then asked how their behavior changed. The answer choices in each domain enabled the generation of the dependent dichotomous variable: experience a negative change (yes/no). A negative change in PA included training for less time or having stopped training completely. A negative change in body weight was defined as weight gain, while a negative change in smoking included increased smoking frequency or experiencing a relapse after having previously quit. Appendix A provides further details on the survey questions.

### 2.4. Statistical Analysis

Descriptive statistics were used to examine the sociodemographic and health behavior characteristics of the total sample based on the self-classification to COVID-19 risk groups (HRG/other). The data are represented as numbers and percentages in parentheses (%) for categorical variables or means and standard deviations (SD) for continuous variables. Chi-square tests were employed for categorical variables, and *t*-tests were employed for continuous variables to compare the sociodemographic and health behavior characteristics and changes in health behaviors between HRG and not high-risk. Logistic regression models were performed to compute the unadjusted and adjusted odds ratios (OR) with 95% confidence intervals (CIs). The univariate model examined the crude association between the self-classification to a COVID-19 risk level and a negative change (yes/no) for each health behavior domain. In order to point at independent characteristics associated with the self-classification to a COVID-19 risk level, we further used a multivariate logistic model and adjusted for potential confounding factors, i.e., for personal and health behavior characteristics that were found to differ significantly among the COVID-19 self-classification groups. All tests were 2-sided and used a significance level of 0.05. Statistical analyses were performed using SPSS statistical software version 27.0 (IBM Corp., Armonk, NY, USA).

## 3. Results

### 3.1. Descriptive Characteristics

The general characteristics of the 1670 included respondents (8.7% response rate) are presented in Table 1. More than half of the respondents (57.4%) were women. The mean age for the total sample was 71.0 ± 4.5 years, with no significant age difference between men and women. Over sixty-five percent of respondents were from the central regions of the country. The majority (78.3%) of respondents classified themselves as COVID-19 HRG. The HRG were slightly older than those in the other group (mean age 71.2 ± 4.5 vs. 70.2 ± 4.7, *p* < 0.001).

### 3.2. Changes in Health Behaviors since the Outbreak of COVID-19

The sample included individuals who perform a relatively high level of physical exercise, with an average of 4.2 ± 2.2 h of exercise per week. Smokers constituted 4.7% of respondents. The majority of the respondents (80.8%) reported that they plan to return to exercise at the health club after the pandemic. Table 2 presents the distribution of changes in health behaviors between March 2020 and the beginning of August 2020. PA was identified with the highest proportion of any change in health behaviors (negative or positive), with 60.5% of participants changing their exercise habits. In addition, the most prevalent negative change reported was also in the amount of PA; 57.4% of the sample reported training less (35.2%) or completely stopped training (22.2%). Over a quarter of the sample (26.6%) reported that they experienced weight gain, with an average of 2.8 ± 1.5 kg gained. Twelve percent reported weight loss, with an average of 4.0 ± 3.1 kg lost. Among current smokers (*n* = 79), 17.7% of the respondents reported a negative change in smoking habits. Twelve participants reported smoking more (*n* = 12), while two participants reported that they had relapsed after having previously quit.

### 3.3. Associations between Changes in Health Behaviors and Self-Classification of COVID-19 Risk Level

#### 3.3.1. Changes in Physical Activity

**Figure 1:**
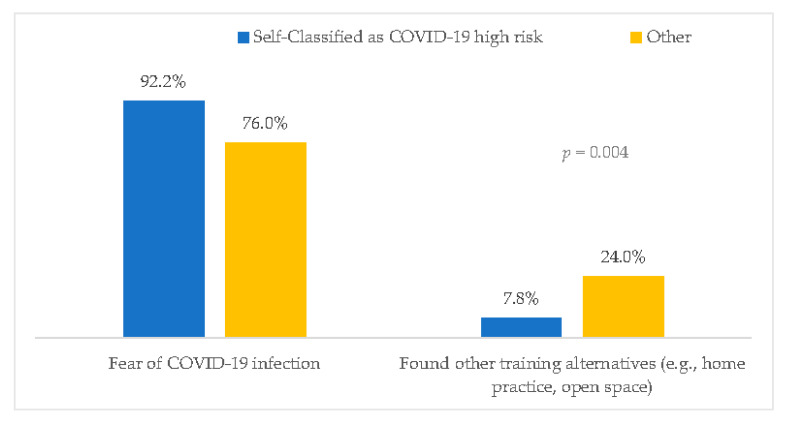
Reported reasons of 321 health club members for not intending to return working out at the club after the closure, according to risk group for COVID-19.

Those who self-classified as COVID-19 HRG were somewhat less active (mean weekly hours of exercise before the pandemic 4.0 ± 2.1 vs. 4.8 ± 2.5, *p* < 0.001) and were more likely to report that they do not intend to return to working out in the health club after the closure than those who were not high-risk (22.0% vs. 9.4%, *p* < 0.001). Of the 321 (19.2%) individuals who do not intend to return to the health club, those who self-classified as HRG were more likely to state fear of contracting the virus as the primary reason compared to those who self-classified as not high-risk (92.2% vs.76%, *p* = 0.004) (Figure 1). Furthermore, this group was more prone to experience a negative change in PA than those who were not high-risk (59.9% vs. 48.2%, *p* < 0.001), as shown in Table 2. No difference in positive change between the two groups was observed.

Table 3 presents the results from regression models for factors associated with negative health behavior changes. The unadjusted logistic models revealed that self-classification to the HRG was associated with 84% greater odds of a negative change in exercise habits (95%CI: 1.41–2.40). In the adjusted multivariate model, the odds of a negative change in PA for those in the HRG compared to the other group were attenuated but still significant (OR = 1.46, 95%CI: 1.10–1.93; *p* = 0.009). In addition, older adult club members who reported that they do not intend to return to the club were much more likely to have reduced their weekly hours in sports activity (OR = 7.60, 95%CI: 5.17–11.16; *p* < 0.001) than those with plans to return. Moreover, each additional hour of weekly exercise performed in the period prior to the pandemic was associated with 7% lower odds of negative behavior (OR = 0.93, 95%CI: 0.88–0.98; *p* = 0.012). Age and smoking status did not significantly associate with a reduced amount of PA.

#### 3.3.2. Changes in Body Weight

A quarter of the sample reported gaining weight, with a greater proportion of participants reporting weight gain among the HRG compared to those who were not high-risk (27.9% vs. 21.8%, *p* = 0.004). The unadjusted logistic regression analysis showed that self-classification to the HRG was associated with 67% greater odds of experiencing weight gain (OR = 1.67, 95%CI: 1.21–2.31; *p* = 0.002). In the multivariate model, the odds of gaining weight slightly increased for those in the HRG compared to the other group (OR = 1.74, 95%CI: 1.25–2.42; *p* = 0.009). Adjusting for COVID-19 risk group, weekly hours performing sports, return to the health club, and smoking, each additional year of age was associated with 4% lower odds of gaining weight (OR = 0.96, 95%CI: 0.93–0.99; *p* = 0.03). Weekly hours in sports, the intention to return to training in the health club, and smoking were not found to be independently associated with weight gain.

#### 3.3.3. Changes in Smoking Habits

The prevalence of smokers was higher in the other group compared to the HRG (7.2% vs. 4.1%, *p* = 0.03). The chi-square test showed no statistical significance in changes in smoking habits between the two groups. A multivariate logistic regression analysis revealed that older age was associated with 28% greater odds of a negative change in smoking habits for each additional life-year (OR = 1.28, 95%CI: 1.08–1.61; *p* = 0.04). The COVID-19 risk group, number of weekly hours spent in sports, and the intention to return to training in the health club were not independently associated with negative smoking behavior.

## 4. Discussion

The results of this survey show that the reduction in the amount spent engaging in PA was the most prevalent negative reported health behavior change among active older adults from the beginning of the COVID-19 pandemic in March 2020 until the beginning of August 2020. More than half of the respondents reported that they were less physically active since the outbreak than during normal times. The next most common health change was observed in body weight, with more than one third of the respondents reporting a change in body weight and over a quarter of the sample reporting that they experienced weight gain. Lastly, among current smokers who reported experiencing a change in smoking habits, almost half of them reported an increase in smoking, while the rest reported a decrease.

This study also reveals that those who self-classified as HRG were more prone to exhibit negative changes in exercise habits and weight gain compared to those who were not high-risk. Furthermore, those in the HRG were more likely to report they do not intend to return to working out in the health club after the pandemic, mostly due to fear of contracting the virus.

### Review of the Scientific Literature in Relation to the Findings

While previous studies confirm that restrictions help lower the level of infection, they affect health behaviors among different population groups due to reducing mobility in general and increasing sedentary behavior [9,10,11,12,13,14,15,16,17,18,19,20,21,22,23,37,38]. The results of this study concur with previous studies showing declines in exercise habits due to the pandemic when exercise was both self-reported [12,13,14,15,16,18,19,23,39,40] and tracked with step counters [5,36]. As for the impact of the pandemic on trainees, Dor-Haim et al. [39] found that 70% of the active adult population trained less than their usual routine during the first lockdown in Israel (April 2020). However, the evidence on this topic is mixed, as several studies reported on an increase in the exercise level [10,11,17,20].

When considering the change in PA that has occurred in older adults, Visser et al. [23] reported a similar prevalence to that found in the current study regarding the decrease in PA (48.3–54.3%). This prevalence was higher than other reported findings [34,41]. Furthermore, when compared with the younger cohorts, some findings suggest that older cohorts were less likely to report changes in health behaviors [10,13,15]. The heterogeneity in the published findings regarding changes in PA might potentially be explained by the specific time the survey was conducted as well as the specific population. In addition, the lives of the citizens and health-related behaviors, among other things, were affected by the COVID-19 restrictions and permits that were applied by the government. All of these factors should be taken into account when comparing the results of this study to other literature in the field. Interestingly, among our sample of elderly health club members who generally attained a relatively high level of PA, only three percent reported positive changes in exercise habits, whereas in other studies, the proportion of individuals who experienced a positive change was much greater, from 19% [15] to 25.2% [19]. This gap between the current study and previous studies could be explained by the hypothesis that health club members are used to practicing in gyms, studios, and other indoor sports facilities that were not available during the pandemic. Therefore, they may have had more difficulty finding alternatives and maintaining an active and balanced lifestyle. The government solution for exercise during the COVID-19 pandemic only allowed individual sports to be practiced in public spaces, which were sometimes far away from people’s places of residence. Therefore, it can be assumed that this solution may have not fit the needs of the individuals enrolled in this study. In terms of health policy, governments should carefully consider barriers, such as closing health clubs, which may jeopardize the ability of the older adult population to maintain an active lifestyle. It is very important to find appropriate solutions for outbreak containment, with the understanding that PA in general and particularly among the older adult population is vital for maintaining physical and mental health.

As for the impact of the pandemic on body weight, more than one third of the respondents reported a change in body weight after almost five months from the beginning of the pandemic. Two months after the crisis began, the vast majority of the studies already reported changes in dietary habits [10,12,14,15,16,17] that resulted in weight gain (range 30–55%) [15,16,17,39] or weight loss (range 18–23%) [15,16]. Two studies found a higher proportion of weight gain compared to weight loss, similar to the trend found in the current study [15,16]. Visser and her colleagues found about 20–30% of older adults reported more snacking, more alcohol consumption, and weight gain [23]. Staying at home, more free time alone, the change in daily routine, limited access to fresh healthy products, such as fruits and vegetables, as well as mental consequences from the pandemic, such as stress and anxiety, may be potential causes of these adverse behavior changes [16,17].

Regarding smoking habits, the rate of smokers in the sample was low compared to the general population in Israel (19.8%) [42], which is typical of those who engage in sports [43]. However, it is possible that the low rate of smoking may not allow for enough statistical power to examine differences in risk level between groups. Nonetheless, half of the smokers experienced an increase in smoking frequency since the beginning of the pandemic. Recent studies underline the mental impact of the COVID-19 pandemic on individuals, suggesting that higher stress levels were observed, mainly due to health and economic concerns [44,45]. Therefore, it can be expected that some individuals might perceive smoking as a form of stress relief, as reported by the smokers in this study [46]. On the other hand, the other half of the smokers reported a positive change in smoking habits. As far as we know, only one previous study has reported findings regarding a positive change in smoking habits during the pandemic. However, this change was a much smaller proportion than that found in this study. Di Renzo and colleagues found that during the Italian COVID-19 lockdown in April 2020, 3.3% of the smokers quit smoking [17]. This positive effect can be explained by the already accepted evidence that smoking was most likely associated with the negative progression and adverse outcomes of COVID-19 [47].

In this study, we found that those who self-classified to COVID-19 HRG were more vulnerable and were more prone to exhibit negative changes in exercise habits and weight gain compared to those who were not high-risk. It can be assumed that those who self-classified to the other group either did not know that they were in a risk group or they did not perceive themselves to be in a high-risk group. The latter can be explained by the concept of ‘risk perception’. Risk perception refers to the individual’s perceived susceptibility to a threat and is an important factor in influencing risk behaviors [4]. Studies that investigated the risk perception amid growing epidemics, such as the 2003 severe acute respiratory syndrome (SARS) epidemic, suggest that a higher perceived risk of infection was associated with engagement in more precautionary behaviors and better compliance with infection control policies [48,49,50]. Iorfa and his colleagues reported a similar finding when focusing on perceived COVID-19 risk [51]. This could be a potential explanation for the findings in this study, as it is possible that those who classified themselves into the HRG were afraid to leave their homes, adopted more preventive behaviors, and as a result, engaged in less PA. It is also plausible that the decline in PA led to weight gain.

Studies have shown that risk perception during an epidemic can be influenced by several factors, including knowledge of the disease, information sources, and emotional states [52]. One study suggests that risk perception mediated the association between COVID-19 knowledge and preventive behaviors [51]. From a public health perspective, this offers a good starting point for risk communication strategies through campaigns or education interventions tailored for elderly individuals in the context of maintaining an active and healthy lifestyle during a pandemic.

The study has several limitations that should be noted. First, the data collected were cross-sectional and the self-classification of respondents to a COVID-19 risk level was determined based on the answer to a single question. This could negatively affect the accuracy of the classification, causing information bias. In addition, although the questions in the survey referred to a change in health behaviors since the outbreak of COVID-19, the possibility of recall bias should be considered. Second, a self-reported questionnaire may have led to social desirability bias, a well-known bias in health behavior surveys. However, the survey was anonymous, which may have reduced the bias to some extent. Third, as in any survey, all of the respondents volunteered to respond, so there may be a selection bias, which limits the generalizability of the study. The above-mentioned biases are typical to online surveys [53]. However, the online format provided us the opportunity to address a large sample from different geographical areas in a short time. It is also worth noting that the response rate to the survey was relatively low. This could be due to the period in which the survey was distributed during the second wave of the pandemic in Israel. People may have been anxious and less mentally prepared to comply with a survey, potentially resulting in selection bias beyond the aforementioned ones. Moreover, the survey did not include questions regarding the level of education, economic status, or other socio-demographic characteristics that are known to be associated with health behaviors [54]. However, all participants were members of the same nationwide health club chain, which has a targeted clientele of middle-class individuals. These individuals choose to spend their leisure time performing social and physical activities in the same environment, increasing the likelihood that the participants hail from a relatively homogeneous background. A comparison of the sex and age distributions between the study sample and the capture population showed a 4.4% higher female proportion in the study sample (*p* < 0.001) but no age differences (*p* = 0.21) (see Table S1). Women have been previously reported to be more responsive than men to online surveys [55]. Finally, it is important to address the definition of a negative change in body weight. The assumption in the study was that weight gain is a negative change; however, we know that this is not necessarily the case for all participants. In addition, we chose not to define weight loss as a positive change because losing weight can be the result of a mental health condition, a decrease in muscle mass due to lack of exercise, and other unhealthy causes. Hence, the results should be carefully considered in the context of the study’s limitations.

There are several strengths to this study as well, including its large and unique sample size. This is the first study to investigate health behavior change among older health club members who were dramatically affected by the restrictions on health clubs and indoor sports facilities. This trend of adverse changes in health behaviors was found among more “health-conscious” populations, which raises even more concerns for the general population.

## 5. Conclusions

This cross-sectional assessment of health behavior changes due to incremental mobility restrictions and the closure of health clubs showed a negative impact, especially among the self-classification to the COVID-19 HRG, on exercise habits and body weight among older health club members. Decision makers should carefully consider policies that can lead to barriers to maintaining a healthy lifestyle and should develop innovative and tailor-made approaches for opening health clubs, even during a pandemic. Furthermore, this study highlights the need to take into account the risk perception of older adults, as it affects their engagement in health behaviors. During a health crises, public health institutions should develop risk communication strategies through awareness campaigns or educational interventions. Drawing on the indication that people with a high-risk perception tend to behave in a more cautious and preventative manner, a realistic perception of personal risk should be encouraged while considering other health-related behaviors such as PA and the maintenance of body weight. Future studies should address additional characteristics, such as sociodemographic and personality traits, that may differentiate between those who self-classified as COVID-19 HRG compared to not high-risk. This could help to better identify this vulnerable group, which is prone to exhibiting negative changes in health behaviors during stressful times, such as the coronavirus pandemic. The present study focused on risk perception of physically active older adults during the second wave of the pandemic. Therefore, future research could examine factors related to risk perception in different times of the pandemic, such as when immunizations were already available. It would also be interesting to explore what led some of the older adult population to classify themselves as belonging to the ‘not high-risk’ group. Finally, research in the field would benefit from an investigation of additional health behaviors, such as changes in diet and sleep patterns, and whether these behaviors are associated with the self-classification of the COVID-19 risk level.

## Figures and Tables

**Table 1:** Socio-demographic and health behavior characteristics of 1670 physically active older Israeli adult members of a health club chain, according to self-classification to COVID-19 level of risk group.

Characteristic	Total Sample	High-Risk Group	Other	*p*-Value ^a^
**N**	**1670 (100)**	**1307 (78.3)**	**363 (21.7)**	
**Sex**, *n* (%)				0.2
Female	959 (57.4)	739 (56.5)	220 (60.6)	
Male	706 (42.3)	564 (43.2)	142 (39.1)	
Unknown	5 (0.3)	4 (0.3)	1 (0.3)	
**Age (y)**, Mean ± SD	71.0 ± 4.5	71.2 ± 4.5	70.2 ± 4.7	<0.001
Female	70.7 ± 4.3	71.0 ± 4.3	70.1 ± 4.4	0.008
Male	71.3 ± 4.8	71.5 ± 4.7	70.5 ± 5.1	0.02
**Residence**, *n* (%)				0.8
Central regions of Israel	1096 (65.6)	863 (66.0)	233 (64.2)	
Peripheral regions of Israel	528 (31.6)	413 (31.6)	115 (31.7)	
Unknown	46 (2.8)	31 (2.4)	15 (4.1)	
**Weekly hours exercising before the pandemic**, Mean ± SD, Median	4.2 ± 2.2, 3.7	4.0 ± 2.1, 3.7	4.8 ± 2.5, 3.7	<0.001
Unknown	171 (10.2)	123 (9.4)	48 (13.2)	
**Work-out place before the pandemic**, *n* (%)				0.06
Gym	741 (44.4)	606 (46.4)	135 (37.2)	
Studio	498 (29.8)	366 (28.0)	132 (36.4)	
Swimming pool	251 (15.0)	205 (15.7)	46 (12.7)	
Home practice	18 (1.1)	13 (1.0)	5 (1.4)	
Open space	19 (1.1)	15 (1.1)	4 (1.1)	
Unknown	143 (8.6)	102 (7.8)	41 (11.3)	
**Do not intend to return to working out in the health club after the pandemic**, *n* (%)	321 (19.2)	287 (22.0)	34 (9.4)	<0.001
Unknown	185 (11.1)	137 (10.5)	48 (13.2)	
**Smoking**, *n* (%)	79 (4.7)	53 (4.1)	26 (7.2)	0.03
Unknown	57 (3.4)	46 (3.5)	11 (3.0)	

^a^ *p*-value derives from comparison analysis of chi-square for categorical variables and *t*-test for continuous variables between the two groups: self-classified as COVID-19 high-risk and not high-risk. Abbreviations: SD—standard deviation

**Table 2:** Changes in health behaviors of 1670 physically active Israeli elderly members of a health club chain between March and the beginning of August 2020, according to self-classification to COVID-19 level of risk group.

Changes in Health Behavior	Total Sample	High-Risk Group	Other	*p*-Value ^a^
**N**	**1670 (100)**	**1307 (78.3)**	**363 (21.7)**	
	***n (%)***	***n (%)***	***n (%)***	
**Physical activity amount**				
No change	624 (37.3)			
Some change	1010 (60.5)			
Less physical exercise	959 (57.4)	784 (59.9)	175 (48.2)	<0.001
More physical exercise	51 (3.1)	32 (2.4)	19 (5.2)	0.61
Unknown	36 (2.2)			
**Body weight**				
No change	983 (58.9)			
Some change	644 (38.5)			
Gained weight	444 (26.6)	365 (27.9)	79 (21.8)	0.004
Weight loss	200 (12.0)	153 (11.7)	47 (12.9)	0.52
Unknown	43 (2.6)			
**Smoking habits**				
No change	39 (49.3)			
Some change	29 (36.7)			
Increased/relapsed smoking	14 (17.7)	9 (17.0)	5 (19.2)	0.62
Reduced smoking	15 (19.0)	11 (20.7)	4 (15.3)	0.82
Unknown	11 (13.9)			

^a^*p*-value derives from chi-square analysis in experiencing a negative change (yes/no) in each one of the three health behaviors between the two groups: self-classified as COVID-19 high-risk/not high-risk.

**Table 3:** Logistic regression models for factors associated with negative health behavior changes (yes/no) from the beginning of the COVID-19 outbreak in March until the beginning of August 2020 among physically active elderly members of the health club chain.

	Variable (Reference Group)	Negative Health Behavior Change		
		Physical Activity *n* = 1570	Weight Gain *n* = 1489	Smoking *n* = 79
		OR (95%CI)	OR (95%CI)	OR (95%CI)
**Univariate** **Regression**	Self-classification to COVID-19 higher risk (no)	1.84 (1.41–2.40)	1.67 (1.21–2.31)	0.70 (0.17–2.77)
**Multivariate Regression**	Self-classification to COVID-19 higher risk (no)	1.46 (1.10–1.93)	1.74 (1.25–2.42)	1.21 (0.25–5.80)
	Age (1-year increment)	1.01 (0.98–1.04)	0.96 (0.93–0.99)	1.28 (1.08–1.61)
	Weekly hours exercising before the pandemic (1-h increment)	0.93 (0.88–0.98)	0.96 (0.91–1.02)	0.53 (0.05–5.48)
	Return to the sport club after the pandemic (yes)	7.60 (5.17–11.16)	0.81 (0.60–1.11)	0.69 (0.42–1.12)
	Smoking (no)	1.12 (0.65–1.92)	1.04 (0.59–1.86)	-

Abbreviations: CI = confidence interval.

## Data Availability

The data presented in this study are available on request from the corresponding author.

## Supplementary Materials

The following supporting information can be downloaded at: https://www.mdpi.com/article/10.3390/ijerph19127039/s1, Table S1. Comparison of sex and age distribution between the study sample and the total older adult population of members of a health club chain.

## Appendix A. Survey Questions and Response Options

**Table A1:** Survey Categories, Questions, and Response Options.

Category	No. of Questions	Questions	Response Options	Remarks
1. Self-classification of COVID-19 risk level	1	“Are you at higher risk of developing serious illness from COVID-19 based on the WHO criteria? Individuals who are considered part of a COVID-19 high-risk group have at least one of the following criteria: age 65 years and older, cardiovascular disease, diabetes, respiratory diseases, diseases affecting the immune system, cancer, smoking, or obesity”.	Yes or no	This question enabled us to divide the sample into two groups by self-classification to COVID-19 risk level and to generate the main independent variable: the high-risk group (HRG) and the not high-risk group, called “Other”. Those who answered “no” were called “other”, as they either do not know that they are in a risk group or they do not perceive themselves as high-risk.
2. Health behaviors	4	a. “How many times a week did you usually exercise (before the pandemic)?” b. “What was usual duration of each workout (in minutes)?” c. “What was your main workout location before the pandemic?” d. “Do you currently smoke?”	a. Number of times b. Duration in minutes c. Gym; studio; swimming pool; home practice; open space d. Yes or no	Questions a and b were calculated to create the variable of ‘weekly hours exercising before the pandemic’ by multiplying the frequency and the duration of exercise and dividing the product into 60 min to create a continuous variable.
3. Changes in health behaviors since the beginning of the COVID-19 outbreak	6	These questions addressed changes in health behaviors for three lifestyle domains: physical activity, body weight, and smoking. Physical activity: a. “Since the coronavirus outbreak, have you noticed any change in your exercise habits?” b. If a respondent answered “yes”, s/he was then asked: “How has it changed?” Body weight: c. “Since the coronavirus outbreak, have you noticed any change in your body weight?” d. If a respondent answered “yes”, s/he was then asked: “How has it changed?” how many kg of weight did you gain or lose? Smoking habits: e. “Since the coronavirus outbreak, have you noticed any change in your smoking habits?” f. If a respondent answered “yes”, s/he was then asked: “How has it changed?”	Physical activity: a. Yes or no b. I’ve been training more than I’m used to; I’ve been training less than I used to; I’ve stopped training Body weight: c. Yes or no d. I’ve gained weight; I’ve lost weight Smoking habits: e. Yes or no f. I’ve been smoking more than I’m used to; I’ve been smoking less than I used to; I’ve relapsed after quitting; I’ve stopped smoking	Questions b, d, and f created the dependent dichotomous variable for each domain: experience a negative change (yes/no). A negative change in physical activity included training less or stopping training, a negative change in body weight included weight gain, and a negative change in smoking included smoking more or a relapse after quitting. A positive change in physical activity included training more, and positive change in smoking included smoking less or stopping smoking. Note: weight loss was not defined as a positive change, as losing weight can be the result of a mental health condition, a decrease in muscle mass due to a lack of exercise, and other unhealthy causes.
4. Returning to exercise at the health club	2	a. “Do you intend to return working out at the health club after the pandemic?” b. If a respondent answered “no”, s/he was then asked: “Why not?”	a. Yes or no b. I’m afraid of getting infected with coronavirus; I have found alternatives that suit me (e.g., internet platform or outdoor activities). A participant could select more than one answer.	
