# Supplementary material for: Association between Self-Classification of COVID-19 Risk Levels and Adverse Lifestyle Changes among Physically Active Older Adults Following the Coronavirus Outbreak
Source: Int J Environ Res Public Health. 2022 Jun 8;19(12):7039. doi: 10.3390/ijerph19127039 (PMC9222379; doi:10.3390/ijerph19127039)
Supplement: Supplementary file 1 [file ijerph-19-07039-s001.zip › ijerph-1698644-supplementary.pdf]

Supplementary Materials

Table S1. Comparison of sex and age distribution between the study sample and the total older adult population of members of a health club chain.

**Characteristic**	**Study sample**	**All older adult (age ≥ 65 years) health club chain members**	***p*- Value**
**N**	**1,670**	**19,160**	
**Sex,** *n (%)*			<0.001
Female	959 (57.4)	10,154 (53.0)	
Male	706 (42.3)	9,006 (47.0)	
**Age (y),** Mean ± SD	71.0 ± 4.5	71.8 ± 5.2	0.21
